# Prioritizing river basins for nutrient studies

**DOI:** 10.1007/s10661-023-12266-7

**Published:** 2024-02-09

**Authors:** Anthony J. Tesoriero, Dale M. Robertson, Christopher T. Green, J. K. Böhlke, Judson W. Harvey, Sharon L. Qi

**Affiliations:** 1 U.S. Geological Survey, Portland, OR USA; 2 U.S. Geological Survey, Madison, WI USA; 3 U.S. Geological Survey, Moffett Field, CA USA; 4 U.S. Geological Survey, Reston, VA USA; 5 U.S. Geological Survey, Lakewood, CO USA

**Keywords:** Monitoring design, Nutrients, Basin selection, Water quality, Federal research, Hydrology

## Abstract

Increases in fluxes of nitrogen (N) and phosphorus (P) in the environment have led to negative impacts affecting drinking water, eutrophication, harmful algal blooms, climate change, and biodiversity loss. Because of the importance, scale, and complexity of these issues, it may be useful to consider methods for prioritizing nutrient research in representative drainage basins within a regional or national context. Two systematic, quantitative approaches were developed to (1) identify basins that geospatial data suggest are most impacted by nutrients and (2) identify basins that have the most variability in factors affecting nutrient sources and transport in order to prioritize basins for studies that seek to understand the key drivers of nutrient impacts. The “impact” approach relied on geospatial variables representing surface-water and groundwater nutrient concentrations, sources of N and P, and potential impacts on receptors (i.e., ecosystems and human health). The “variability” approach relied on geospatial variables representing surface-water nutrient concentrations, factors affecting sources and transport of nutrients, model accuracy, and potential receptor impacts. One hundred and sixty-three drainage basins throughout the contiguous United States were ranked nationally and within 18 hydrologic regions. Nationally, the top-ranked basins from the impact approach were concentrated in the Midwest, while those from the variability approach were dispersed across the nation. Regionally, the top-ranked basin selected by the two approaches differed in 15 of the 18 regions, with top-ranked basins selected by the variability approach having lower minimum concentrations and larger ranges in concentrations than top-ranked basins selected by the impact approach. The highest ranked basins identified using the variability approach may have advantages for exploring how landscape factors affect surface-water quality and how surface-water quality may affect ecosystems. In contrast, the impact approach prioritized basins in terms of human development and nutrient concentrations in both surface water and groundwater, thereby targeting areas where actions to reduce nutrient concentrations could have the largest effect on improving water availability and reducing ecosystem impacts.

**Supplementary Information:**

The online version contains supplementary material available at 10.1007/s10661-023-12266-7.

## Introduction

Nutrients are essential for ecosystem health, but in excess can have detrimental effects on human health and the ecology of surface-water systems. Since the beginning of industrialization, human activities, particularly crop production and wastewater discharges, have greatly altered the amount and distribution of nutrients across terrestrial and aquatic biospheres. For example, anthropogenic activities have roughly doubled the amount of reactive nitrogen (N) that enters the terrestrial biosphere since industrialization (Smil 1999; Galloway et al. 2008) and redistributed phosphorus (P) across the globe because of mining and fertilizer use (Gilbert 2009). In the United States (U.S.), total N inputs and surpluses remain dominated by agricultural processes (Sabo et al. 2019). As world population and food demand continue to increase, so too has the delivery of N and P to terrestrial and aquatic ecosystems, with potentially far-reaching implications for ecosystems and human health (Beusen et al. 2022, U.S. Environmental Protection Agency 2011).

Changing the distribution and amount of N and P in the environment has led to a wide range of negative environmental impacts (Erisman et al. 2013). These impacts include the degradation of drinking water supplies (Pennino et al. 2020) with increasing human health concerns (Ward et al. 2018), increased eutrophication and harmful algal blooms (HABs) (Boesch 2002; Rabalais 2002; Schindler et al. 2016), contributions to climate change (Thompson et al. 2019; Fagodiya et al. 2017), and biodiversity loss (Finzi et al. 2011).

Given the broad scope of the impacts of nutrient enrichment on ecosystems and human health, a systematic, scientifically defensible approach to prioritize areas to study and address nutrient-driven water-quality issues (Harvey et al. 2023; Tesoriero et al. 2023) may support management decision-making. Previous efforts to prioritize scientific studies of water resources at a large regional scale or the scale of the contiguous U.S. (CONUS) have been based on either measured or predicted water quality (Robertson et al. 2009) or variables that broadly affect water availability (Van Metre et al. 2020). Prioritization of CONUS basins with respect to specific water-quality issues may benefit from holistic and adaptable approaches that encompass the extreme variations of sources and processes affecting nutrient concentrations across aquatic ecosystems and water supplies. The most successful basin prioritization strategies may be those that identify basins that could most benefit from improved nutrient management strategies as well as basins that may be best suited for measurement and modeling studies to improve scientific understanding.

National programs have used different approaches to select sites and areas for study to assess nutrient concentrations in surface-water and groundwater resources in the U.S. In 1990, the U.S. Environmental Protection Agency (USEPA) began the Environmental Monitoring and Assessment Program (EMAP) focusing on the status and trends of ecological resources of surface waters of the U.S. (U.S. Environmental Protection Agency 2016). The EMAP program was succeeded by the USEPA National Rivers and Streams Assessment (U.S. Environmental Protection Agency 2020), with both programs collecting data, at a limited frequency, from randomly chosen stream sites throughout the U.S. In 1991, the U.S. Geological Survey (USGS) launched the National Water Quality Assessment (NAWQA) Program (Leahy et al., 1990) with similar goals as EMAP, but NAWQA also included groundwater. NAWQA’s approach was to collect more intensive water-quality data from 60 mid-sized basins (study units) to understand the factors affecting water quality and to support the development of water-quality models. The 60 NAWQA study units were chosen to cover a large fraction of the Nation’s freshwater use, the major hydrologic regions, and a diversity in water-quality stressors (Gilliom, Alley and Gurtz 1995).

**Fig. 1:**
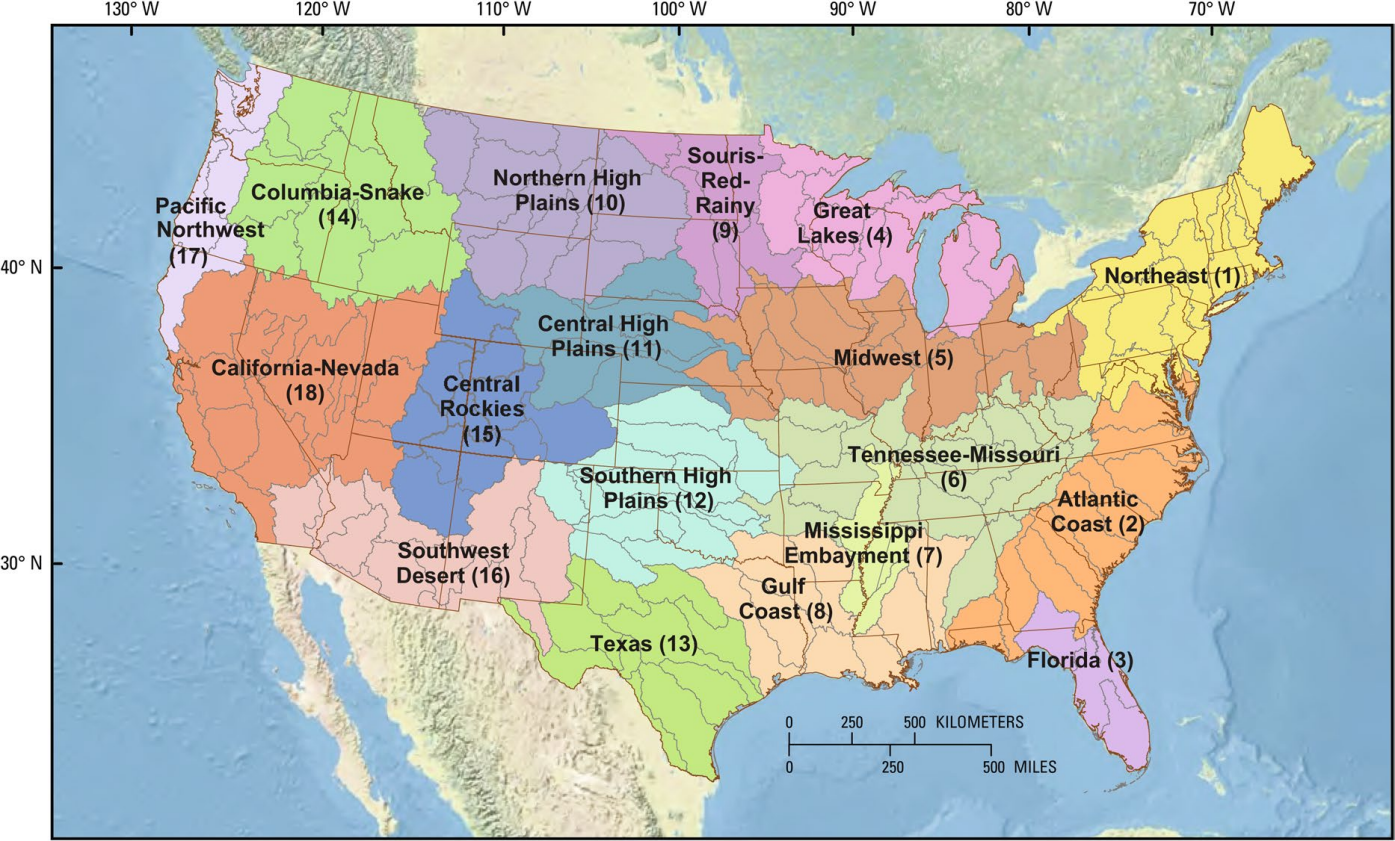
The 163 candidate basins overlain with the 18 hydrologic regions as defined by Van Metre et al. (2020). The numbers in parentheses after the region names are used to identify regions in subsequent figures

More recently, a quantitative approach to prioritize candidate basins for monitoring investment was developed to understand changes in water availability and advance the objectives of new USGS programs (Van Metre et al. 2020). In the Van Metre study, the CONUS was divided into 18 regions (referred to as “hydrologic regions or HRs”), each with relatively homogeneous hydrologic drivers and processes to represent the wide diversity in conditions that exist across the CONUS (Fig. 1). Candidate basins within the HRs were developed by Van Metre et al. (2020) based on the 203 level-4 hydrologic units (HUC04) (Seaber, Kapinos and Knapp 1987) within the CONUS. Some of the smaller HUC04s were combined to reduce the range of basin sizes, resulting in 163 final candidate “HUC04-sized” basins (Fig. 1). Basins within each HR were then ranked based on 10 geospatial variables that focused on the potential impact of anthropogenic stressors and the importance of basin water resources to ecosystems and human health (receptors). Stressors included land use, climate change, and fire risk. Variables describing the importance and state of basin water resources included water-balance components and ecosensitivity. Selecting basins from as many of the HRs as possible would ensure that the selected basins represent a wider range of major hydrologic landscape conditions in the CONUS (Van Metre et al. 2020).

The purpose of this work is to develop strategies to rank the suitability of basins for studies of nutrient (N and P) effects on water availability. The approaches developed in this study build on a previous study (Van Metre et al. 2020), which ranked basins primarily on water quantity and sustainability, but did not specifically focus on water quality. In the current study, variables for ranking approaches were selected based on existing data, models, and key issues and research questions related to the occurrence and effects of nutrients in each region and nationally. HUC04-sized basins were ranked with respect to (1) urgency of nutrient-related water-quality problems and (2) opportunity to improve understanding of nutrient transport processes and water-quality predictive skill. Maps were developed for both national and regional prioritization. Prioritization scenarios were compared to evaluate differences and to highlight key issues affecting optimal basin-selection for understanding of the effects of nutrients (N and P) on ecosystems and human health.

## Approach

Two approaches were applied to rank basins: one similar to a previously developed method (Van Metre et al., 2020), but focused on identifying those basins most at risk to the impacts of nutrients on ecosystems and human health; and a second approach focused on identifying those basins that are best suited for studies that seek to understand the drivers affecting nutrient concentrations and related effects on environmental conditions. All variables used in both ranking approaches are publicly available in the data release associated with this article (Qi et al. 2023). The first approach ranks basins based on their likelihood to be impacted by excess nutrients, either now or in the future. This approach relies on variables describing predicted nutrient sources, stream and groundwater nutrient concentrations, and potential impacts on several receptors. This approach is termed the “impact approach.”

The second ranking approach is like the impact approach in that it includes variables describing predicted concentrations of nutrients in streams, nutrient sources, and receptors, but also places high importance on ranges in nutrient concentrations and the factors affecting nutrient concentrations. This second approach is termed the “variability approach.” A range in stream nutrient concentrations and the potential factors affecting nutrient concentrations may better enable a gradient of nutrient impacts to be examined and may be better suited to describe the processes responsible for the observed impacts on ecosystems and drinking water supplies.

Ranking assessments were conducted at a CONUS scale and a regional scale using the 18 hydrologic regions (HRs) defined in a previous study (Van Metre et al. 2020). The HRs were delineated to maximize the homogeneity of major hydrologic drivers and processes within each region. The HRs delineated the physical and climatic setting of the CONUS using land-surface form, geologic texture (permeability of soil and bedrock), and climate variables (Wolock, Winter and McMahon 2004). HRs were delineated using *k*-means clustering in the CONUS based on hydrologic landscape region proportions and the coordinates of the centroid of each candidate basin (Van Metre et al. 2020) (Fig. 1). These delineations resulted in relatively homogeneous physical and climatic characteristics within each of the 18 HRs, while the differences among regions capture the national diversity in temperature, precipitation, elevation, and other major drivers of the hydrologic cycle. The numbering system for the HRs progresses from northeast to southwest across the CONUS, HR1 for the Northeast to HR18 for California-Nevada (Fig. 1).

### Impact ranking approach

The impact approach relied on 14 variables (Qi et al. 2023) representing nutrient *water quality* in groundwater and streams, *sources* of nutrients, and current or potential effects on *receptors*, such as ecosystems and human health (Table 1). While each of these three categories are interrelated, it was considered important to subdivide them to capture those instances where these interrelations were affected by other factors. For example, geochemical and hydrologic factors may affect the degree to which large nutrient sources result in high nutrient concentrations in streams (Wherry, Tesoriero and Terziotti 2021) and groundwater (Tesoriero, Stratton and Miller 2021). Similarly, the societal impact of large sources and high nutrient concentrations may vary depending on the characteristics of the receptor (e.g., population density, proximity to receiving waters).

**Table 1:** Variables used in the impact approach

Variables	Category	Units	Weighting factor	Reference
N and P from manure and fertilizer	Source	kg/km^2^/year	1 for both N and P	(Wieczorek, Jackson and Schwarz 2018)
N and P from wastewater treatment plants	Source	kg/km^2^/year	1 for both N and P	(Wieczorek et al. 2018)
N from septic systems	Source	kg/km^2^/year	1	(LaMotte 2018)
Predicted nitrate concentration in shallow groundwater	Water quality	mg/L as N	1.5	(Nolan and Hitt 2006)
Predicted nitrate concentration in groundwater at the depth of public supply	Water quality	mg/L as N	0.5	(Ransom et al. 2022)
Median predicted N and P concentration in streams (median of HUC08 basins)	Water quality	mg/L	1.0 for both N and P	(Ator 2019, Robertson and Saad 2019, Wise, Anning and Miller 2019, Wise 2019, Hoos and Roland 2019)
Population served by public and domestic supplies	Receptor	People/km^2^	1	(Johnson, Belitz and Lombard 2019, Johnson et al. 2022, Johnson et al. 2021)
Percent waterbodies	Receptor	% open water	1	(Dewitz and U.S. Geological Survey 2021)
Biotic index	Receptor	Likelihood stream is not in good biological condition	1	(Hill et al. 2017)
N and P delivered to receiving waters	Receptor	kg/km^2^/year	0.5 for both N and P	(Ator 2019, Robertson and Saad 2019, Wise et al. 2019, Wise 2019, Hoos and Roland 2019)

Note that N and P variables were included in the ranking approach as separate variables. See Supplemental information and Qi et al. (2023) for more details

Water quality was considered by using four variables that represent N and P concentrations in streams and groundwater. Model-estimated stream concentrations of N and P were chosen to describe the most impacted areas of the surface water system. For each candidate HUC04-sized basin, concentrations of N and P in streams were described using the median predicted concentration among the HUC08 sub-basins in each HUC04-sized area. Each HUC08 concentration was estimated using predicted volumetrically weighted-mean (VWM) N and P concentrations of the most downstream reach (the reach with the highest estimated area after correcting for diversions) in each HUC08 sub-basin from regional SPARROW watershed models that covered the CONUS (Wise 2019, Hoos and Roland 2019, Wise et al. 2019, Ator 2019, Robertson and Saad 2019). Model-estimated nitrate concentrations in shallow groundwater (Nolan and Hitt 2006) were selected to represent the most impacted areas of the groundwater system and to represent the potential impact of groundwater discharge of N to streams. Predicted nitrate concentrations at the depth of public supply wells (Ransom et al. 2022) were selected to represent the deeper portions of aquifer systems and to represent the relative risk to public drinking water supplies of elevated nitrate concentrations. Groundwater nitrate concentrations in shallow groundwater and at the depth of public supplies were represented by the median of gridded predicted concentration values within each HUC04-sized basin for each depth. Weights of 1.5 and 0.5 in the ranking were applied to variables representing shallow groundwater nitrate and nitrate at the depth of public supply, respectively; greater importance was placed on the shallow portion of the groundwater system because higher nitrate concentrations and greater potential impact on streams are typically found in the shallow portions of aquifers. Increased weight on the shallow portion of the aquifer system also prioritizes private domestic wells as these wells tend to be shallower than public supply wells. Nitrate concentrations tend to be higher in shallow groundwater in agricultural areas and in private wells than in other land use or well types (Burow et al. 2010). An increased emphasis on shallow groundwater is also appropriate since water withdrawn from shallow private wells is less likely to be tested and treated than water from public supply wells (Zheng and Flanagan 2017).

Five variables were chosen to represent the major anthropogenic sources of N and P in the hydrologic cycle across the CONUS: N from manure and fertilizer applied to cropland, P from manure and fertilizer applied to cropland, N from wastewater treatment plants discharged to streams, P from wastewater treatment plants discharged to streams, and N from septic systems (Wieczorek et al. 2018, Skinner and Wise 2019, LaMotte 2018). All source variables were presented as the annual mass applied to the landscape or discharged to a stream, which were then divided by the total area of the basin to normalize sources with respect to basin size (e.g., kg/km^2^/year).

Five variables were selected to represent the major receptors affected by elevated concentrations of N and P. Ecosystems effects were represented in the ranking by a biotic index that predicts the likelihood that a stream would be classified as having good biological condition. These predictions were made by relating USEPA National Rivers and Streams Assessment benthic multimetric index classes to landscape features using random forest classification (Hill et al. 2017). Multimetric indices were calculated using taxonomic data of benthic invertebrates from sampled sites. In the current ranking, preference was given to watersheds with a higher likelihood of not being in good condition. Impacts of basin-derived nutrients on receiving waters outside the basins (e.g., Gulf of Mexico, Great Lakes, and other marine coastal waters) were represented by the sum of the delivered incremental loads estimated from SPARROW (Hoos and Roland 2019, Robertson and Saad 2019, Wise et al. 2019, Wise 2019, Ator 2019, Schwarz et al. 2006) from the HUC04-sized basin to its distant (e.g., coastal) receiving water divided by its total basin area (yield, in kg/km^2^/year). Relative impacts on distant receiving waters can be different from local impacts within basins because of nutrient transformations during transit through larger river networks; for example, N yields delivered to the Gulf of Mexico from two basins with identical internal yields would be lower for the basin farther upgradient from the Mississippi River and the coast (Alexander et al. 2008). N and P yields to distant receiving waters each received a 0.5 weight in this ranking approach to give impact on receiving waters equal importance as the other three receptors in this category (i.e., drinking water, ecosystems, and local waterbodies). The potential impact of nutrients on drinking water sources was described by the total population served from domestic and public supplies (Johnson et al. 2022, Johnson et al. 2021, Johnson et al. 2019) divided by basin area. The potential impact of excess nutrients on lakes and reservoirs was addressed by including percent of basin area classified as open water (Dewitz and U.S. Geological Survey 2021) as a variable.

Individual scores were determined for each of the three main categories (i.e., water quality, sources, and receptors) by ranking each variable across all 163 basins, applying weighting factors for each variable, then summing these weighted ranks for all variables in each category. All 163 basins were used for the overall regional rankings to minimize the effect of small differences in a variable on these rankings. All individual and final rankings were done using the *percentrank* function in Excel (Microsoft Corporation 2023). This category score was then divided by the number of variables, to calculate an overall category score. For example, the water quality score was calculated as follows:

$$Water\:Quality\:Score=\{1.5\times[ Shallow\:Groundwater\:Nitrate\:Rank]+0.5\times[Groundwater\:Nitrate\:at\:PWS\:Depth\:Rank]+1.0\times[Surface\:Water\:N\:Rank]+1.0\times[Surface\:Water\:P\:Rank]\}/4$$

An overall score was determined for each basin by summing the three category scores (see equation below). These scores were ranked within a region to determine regional rankings and across all 163 basins to develop national rankings. Each of the categories were given the same weight.

$$Impact\:Approach\:Overall \:Score=1.0\times[ Water\:Quality\:Score]+1.0\times[Source\:Score]+1.0\times[Receptor\:Score]$$

### Variability ranking approach

In the variability ranking approach, basins were ranked based on their potential to provide information that could improve our understanding of how various factors affect nutrient concentrations in streams and how nutrients affect ecosystems. The variability approach relies on: (1) ranges in N and P concentrations in all of the HUC08s in a HUC04-sized area and the minimum HUC08 N and P concentration in a HUC04-sized area to describe *water quality*, (2) ranges in the *factors* potentially affecting N and P concentrations, (3) the *accuracy* of model predictions of N and P concentrations in streams, and (4) current or potential impacts of nutrients on *receptors* (Qi et al. 2023) (Table 2). Rather than only choosing variables that describe median nutrient conditions and the impact of nutrients (basis behind the impact approach), ranges in N and P concentrations and ranges in the factors affecting N and P concentrations throughout the basin were chosen to prioritize areas. Large ranges in N and P concentrations and factors affecting concentrations within a selected basin may better enable the discernment of empirical and process-driven relations between drivers and stream concentrations (Brett et al. 2005). If the relations can be found, they may then be incorporated into water-quality models that predict stream conditions in unmonitored areas.

**Table 2:** Variables used in the variability approach

Variables	Category	Units	Weighting factor	Reference
Range in predicted N and P concentrations in streams	Water quality	mg/L of N or P (logarithmically transformed)	0.5 for both N and P	(Ator 2019, Robertson and Saad 2019, Wise et al. 2019, Wise 2019, Hoos and Roland 2019)
Minimum predicted N and P concentrations in streams	Water quality	mg/L of N or P (logarithmically transformed)	0.5 for both N and P	(Ator, 2019; Hoos and Roland 2019; Robertson and Saad 2019; Wise 2019; Wise et al. 2019)
Range in N and P from manure and fertilizer application rates	Factor	kg/km^2^/year	0.5 for both N and P	(Wieczorek et al. 2018)
Range in N and P from wastewater treatment plants per unit area	Factor	kg/km^2^/year as N or P	0.5 for both N and P	(Wieczorek et al. 2018)
Range in N from septic systems per unit area	Factor	kg/km^2^	1	(LaMotte 2018)
Range in tile drain density	Factor	Fraction of HUC08	1	(Valayamkunnath et al. 2020)
Range in runoff rates	Factor	m/year	1	(Wieczorek et al. 2018)
Range in predicted nitrate concentrations in shallow groundwater	Factor	mg/L as N	0.5	(Nolan and Hitt 2006)
Range in predicted nitrate concentrations in groundwater at the depth of public supply	Factor	mg/L as N	0.5	(Ransom et al. 2022)
Mean percent residual in N and P predictions	Accuracy	kg/km^2^/year logarithmically transformed	1 for both N and P	(Ator 2019, Robertson and Saad 2019, Wise et al. 2019, Wise 2019, Hoos and Roland 2019)
Total population served by public and domestic supplies per unit area	Receptor	People/km^2^	1	(Johnson et al. 2019, Johnson et al. 2022, Johnson et al. 2021)
Percent waterbodies in basin	Receptor	% open water	1	(Dewitz and U.S. Geological Survey 2021)
Mean biotic index in basin	Receptor	Likelihood stream is not in good biological condition	1	(Hill et al. 2017)
N and P delivered (yield) to downstream receiving waters	Receptor	kg/km^2^/year	0.5 for both N and P	(Ator 2019, Robertson and Saad 2019, Wise et al. 2019, Wise 2019, Hoos and Roland 2019)

All ranges were computed from the 25th and 75th percentile of the HUC08 values. Note that the N and P variables were included in the ranking approach as separate variables. See supplemental information and Qi et al. (2023) for more details

Four variables were used to determine the water quality score, focusing on the variability in nutrient concentrations in streams: the range in the logarithmically transformed VWM N and P concentrations at the most downstream reach of all HUC08 basins (after adjusting for diversions) within each HUC04-sized basin, and the minimum VWM N and P concentrations at the most downstream reach of all HUC08 basins. All VWM concentrations were estimated with regional SPARROW watershed models (Wise 2019, Hoos and Roland 2019, Wise et al. 2019, Ator 2019, Robertson and Saad 2019, Schwarz et al. 2006). All ranges in these and other variables were based on the difference between the 75th percentile value and the 25th percentile value to remove the effects of outliers. The minimum VWM concentrations were chosen as variables because the largest biological response is often found at relatively low nutrient concentrations (Robertson et al. 2006, Wang, Robertson and Garrison 2007).

Nine variables were chosen to prioritize areas based on the range in factors potentially affecting nutrient concentrations in streams. These factors included the range in the following: median predicted groundwater nitrate concentrations at the depth of public supply wells (Ransom et al. 2022); median predicted nitrate concentrations in shallow groundwater (Nolan and Hitt 2006); N and P input from fertilizers and manure (kg/km^2^/year) (Wieczorek et al. 2018); N and P input from wastewater treatment plants (kg/km^2^/year) (Skinner and Wise 2019); percentage of the basin with tile drains (Valayamkunnath et al. 2020); runoff normalized to basin area (m/year) (Wieczorek et al. 2018); and N input from septic systems (kg/km^2^/year) (LaMotte 2018). The range in each variable was determined by the range in values (difference between the 75th percentile value and the 25th percentile to remove the effects of outliers) determined from all HUC08 areas within each HUC04-sized basin.

Two variables were chosen to describe the ability of models to predict nutrient concentrations and loads in streams. This ability was quantified using the mean residuals for estimated loads within each HUC04-sized basin in regional N and P SPARROW models (Wise 2019, Hoos and Roland 2019, Wise et al. 2019, Ator 2019, Robertson and Saad 2019, Schwarz et al. 2006). While other water quality models predicted nutrient concentrations in some areas, model accuracy was based on SPARROW predictability because results from this model were available nationally. Basins with larger residuals (percent error in loads in logarithmic units) were more highly ranked, based on the assumption that more could be learned from new studies in these areas than in areas with small residuals. Model accuracy was included in the variability approach, but not the impact approach, because the variability approach focuses on areas where understanding of nutrient transport processes could most benefit from further study. For the receptor category, the variability approach used the same five variables as the impact approach to describe each HUC04-sized basin’s potential effect of nutrients on local and downstream waters (see “Impact ranking approach” section).

For the variability approach, each basin was first ranked based on each of the 20 variables described above. Ranks were determined within each HR and within the entire CONUS (0 being the least favorable basin and 1 being the most favorable basin). The ranks within each category were then summed to provide four category scores for each HR and the entire CONUS (see Variability Ranking Approach worksheet in supplemental information for more details). Several variables were given a weighting score of 0.5 in determining the general category score, so that a specific characteristic was not overemphasized, including: downstream N and P delivery, groundwater nitrate concentrations at the depth of public supply wells and in shallow groundwater, N and P inputs from fertilizers plus manure, and N and P inputs from wastewater treatment plants. 

To assure that a wide range in nutrient concentrations was observed among subbasins in the final highest ranked HUC04-sized basin and at least one subbasin had low nutrient concentrations, only basins in the top four within its HR with respect to its water quality score were considered for further ranking within a HR. This was done by subtracting 5 points from its water-quality score if the basin was not one of the top four basins in its HR, which basically removed that basin from consideration in that HR. Since the ability of models to simulate nutrient concentrations was based on only one type of model (SPARROW; Schwarz et al. 2006), this variable was not considered as important as categories representing variability in concentrations and factors affecting nutrients concentrations. Therefore, this category was given a 0.5 weight in the overall scoring for both within HR and CONUS scoring. The receptor category was included in the ranking to prioritize areas that had potentially more societal impact from high nutrient concentrations, but because we did not want this information to be as important as the variability in concentrations and factors affecting nutrients concentrations, the receptor category was given a 0.5 weight. The weighted ranks from each of the four categories were then summed into one overall score and then the overall scores were ranked within each HR and the CONUS to provide one overall ranking within each HR and for the CONUS.

$$Variability\:Approach\:Overall\:Score=\{1.0\times[ Water\:Quality\:Rank]+1.0\times[Factors\:Rank]+0.5\times[Receptor\:Rank]+0.5\times[Accuracy\:Rank]\}$$

## Results and discussion

### Ranking for the CONUS

**Fig. 2:**
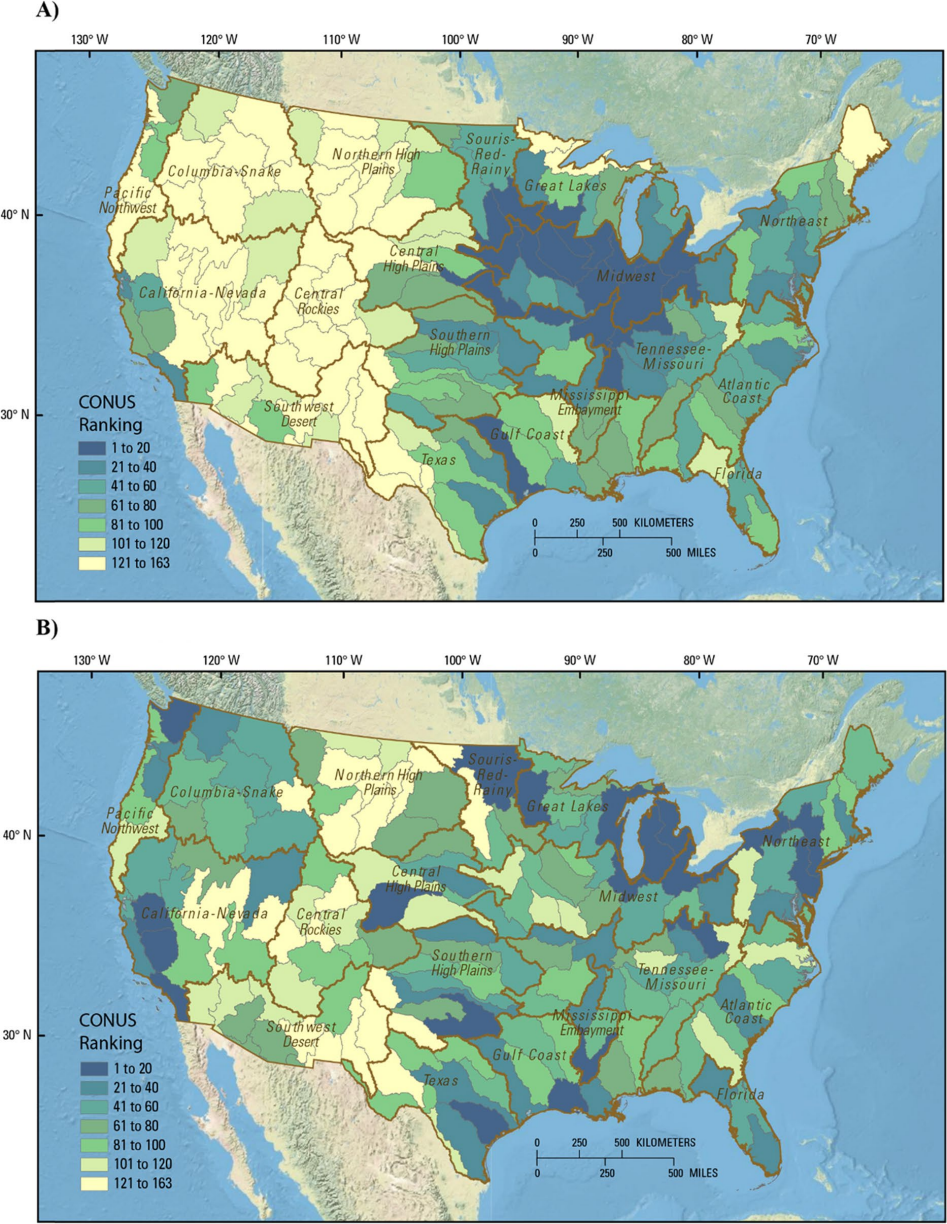
Ranking results for candidate basins throughout the contiguous United States (CONUS) for (A) impact ranking approach and (B) variability ranking approach. Ranking and supporting data are provided in the Supplemental material and in Qi et al. (2023)

The impact and variability approaches were first used to rank basins for the CONUS (Fig. 2). The top selections using the impact approach were concentrated in intensive agricultural areas centered in the Midwest and generally coincided with previous estimates of surplus anthropogenic N (Sabo, Clark and Compton 2021). Four of the top 5 basins were in the Midwest region. Other highly ranked basins were found in the Midwest and Tennessee-Missouri regions as well as in the Souris-Red-Rainy, Gulf Coast, Central High Plains, and Great Lakes regions (Fig. 2, top panel). In contrast, top selections from the variability approach were spread out over the CONUS, especially on the fringes of intense agriculture, north of the corn belt, and north of the agricultural areas of the eastern U.S. (Fig. 2, bottom panel).

**Fig. 3:**
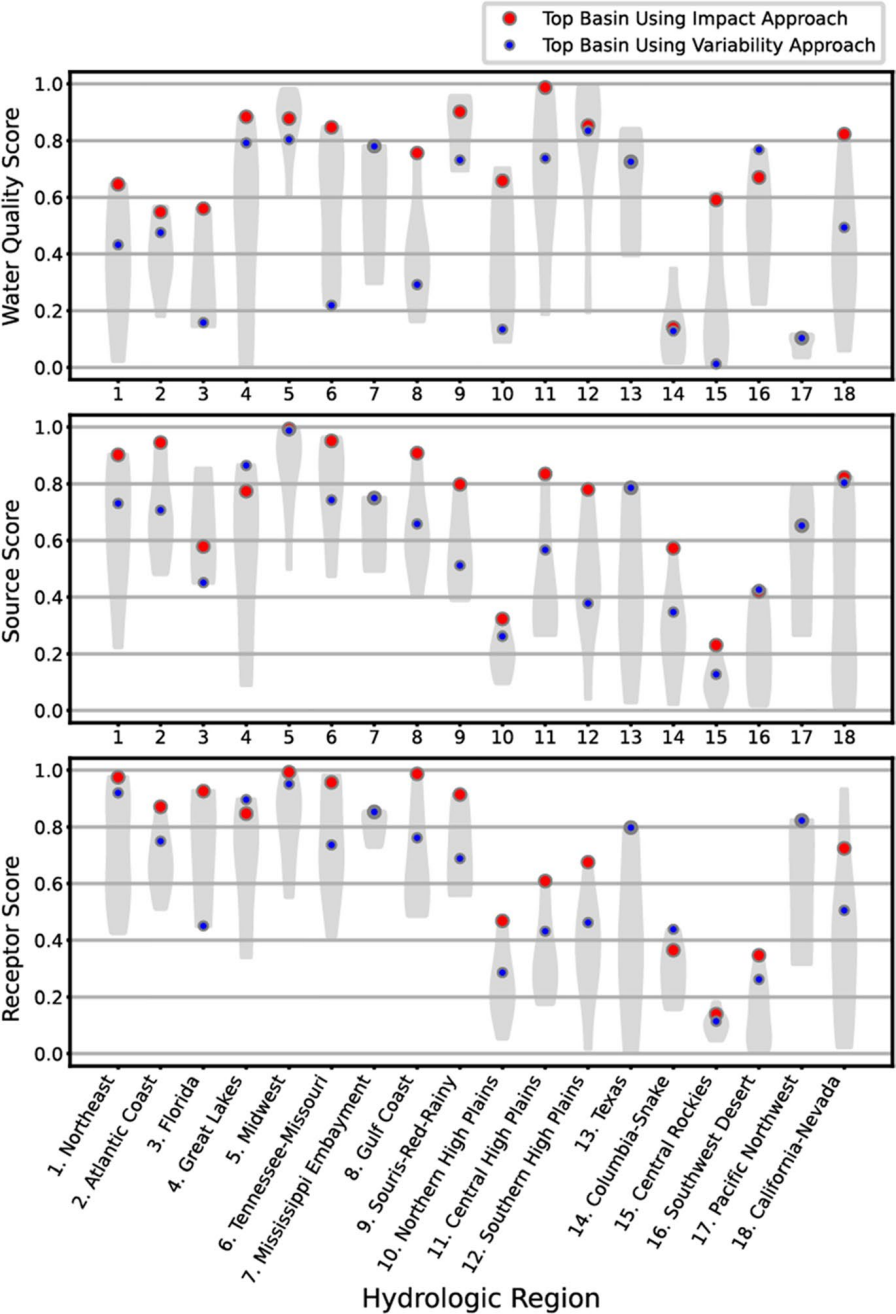
Category scores for the impact approach are shown using violin plots. Category scores range from 0 (least impacted basin) to 1 (most impacted). See text for details. Violin plots depict data distributions using density curves; the width of a curve corresponds to the frequency of data at a point. The overall top-ranked basins in each region for the impact and variability approaches are identified with red and blue dots, respectively

**Fig. 4:**
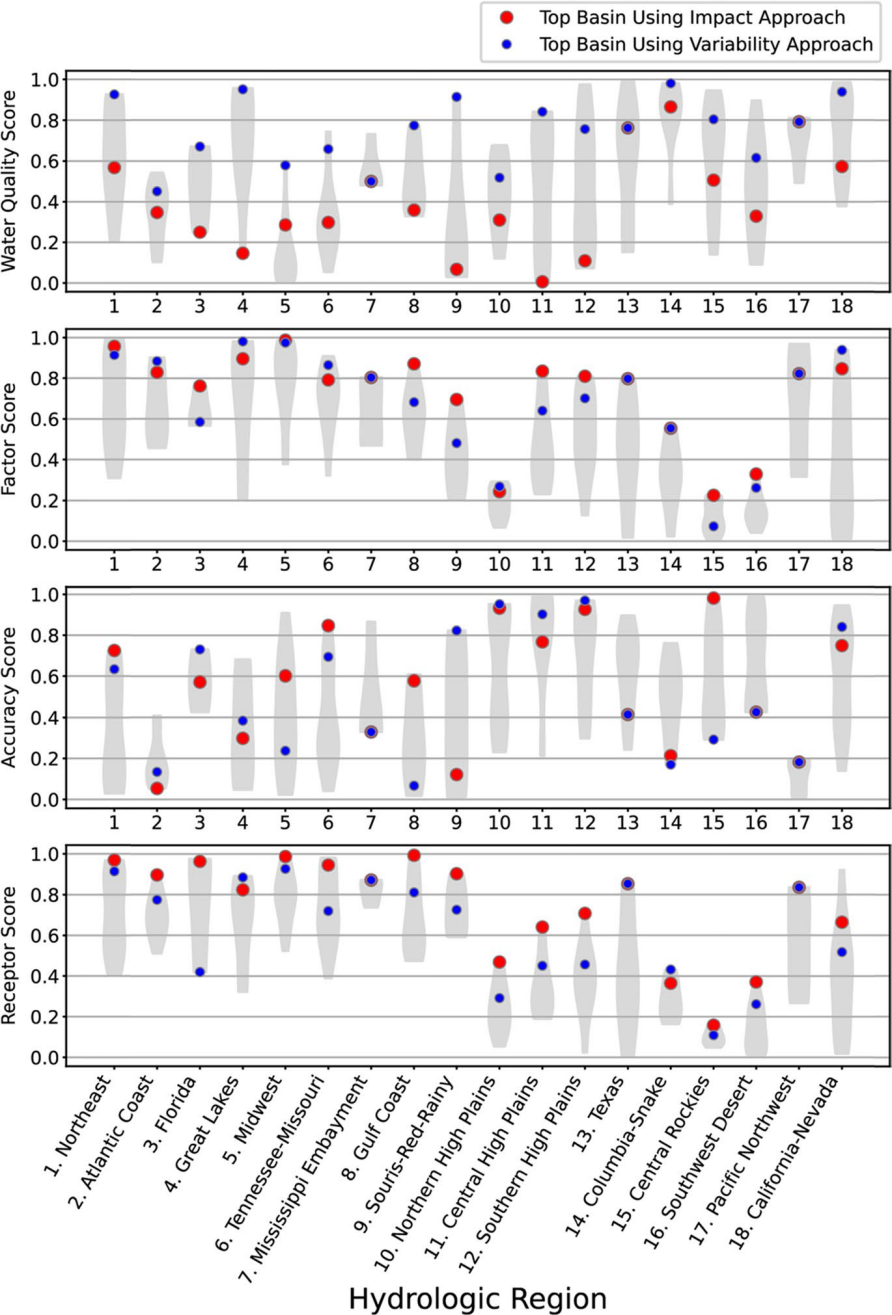
Category scores for the variability approach are shown using violin plots. Category scores range from 0 (least impacted basin) to 1 (most impacted). See text for details. Violin plots depict data distributions using density curves; the width of a curve corresponds to the frequency of data at a point. The overall top-ranked basins in each region for the impact and variability approaches are identified with red and blue dots, respectively

The basin characteristics that most strongly affected rankings were evaluated by examining the scores from each category of variables (e.g., water quality, source, and receptor scores for the impact approach and the water quality, factor, model accuracy, and receptor scores for the variability approach) throughout the CONUS and within each HR. Graphs are provided that show the distribution of the categories nationally for both the impact and variability approaches (Figs. 3 and 4). For the impact approach, the basins that generally had the highest water-quality scores were in the Midwest (HR 5), Souris-Red-Rainy (HR 9), Central High Plains (HR 11), and Southern High Plains (HR 12) regions (Fig. 3, top panel). These regions all have well-documented surface-water (Hanrahan et al. 2018, Van Metre et al. 2016) and/or groundwater-quality degradation (Gurdak, McMahon and Bruce 2011, Scanlon et al. 2010) related to high nutrient concentrations.

High source scores occurred in many areas of the country, such as the Northeast (HR 1), Atlantic Coast (HR 2), Midwest (HR 5), and Tennessee-Missouri (HR 6) regions (Fig. 3, middle panel). Not surprisingly, some HRs with high water-quality scores, like the Midwest (HR 5), also had high source scores. Anomalously high or low concentrations relative to source strength may result from factors related to travel times, transformations, and retention of nutrients within watersheds (Tesoriero et al. 2013, Bohlke 2002, Hill 2019, Sharpley et al. 2013, Liao et al. 2012). For example, in the Midwest, where water-quality and sources scores were high, tile drains are common and are a major pathway for rapid nutrient transport to streams (Pace et al. 2022, Royer, David and Gentry 2006, Schilling et al. 2012, Schilling et al. 2019), thus bypassing nutrient attenuation processes occurring in the riparian zones and groundwater (Hill 2019). Tile drains and other landscape features affecting subsurface flow paths and basin residence times can affect short-term responses as well as decadal trends of nutrient concentrations in streams (Green et al. 2014).

**Fig. 5:**
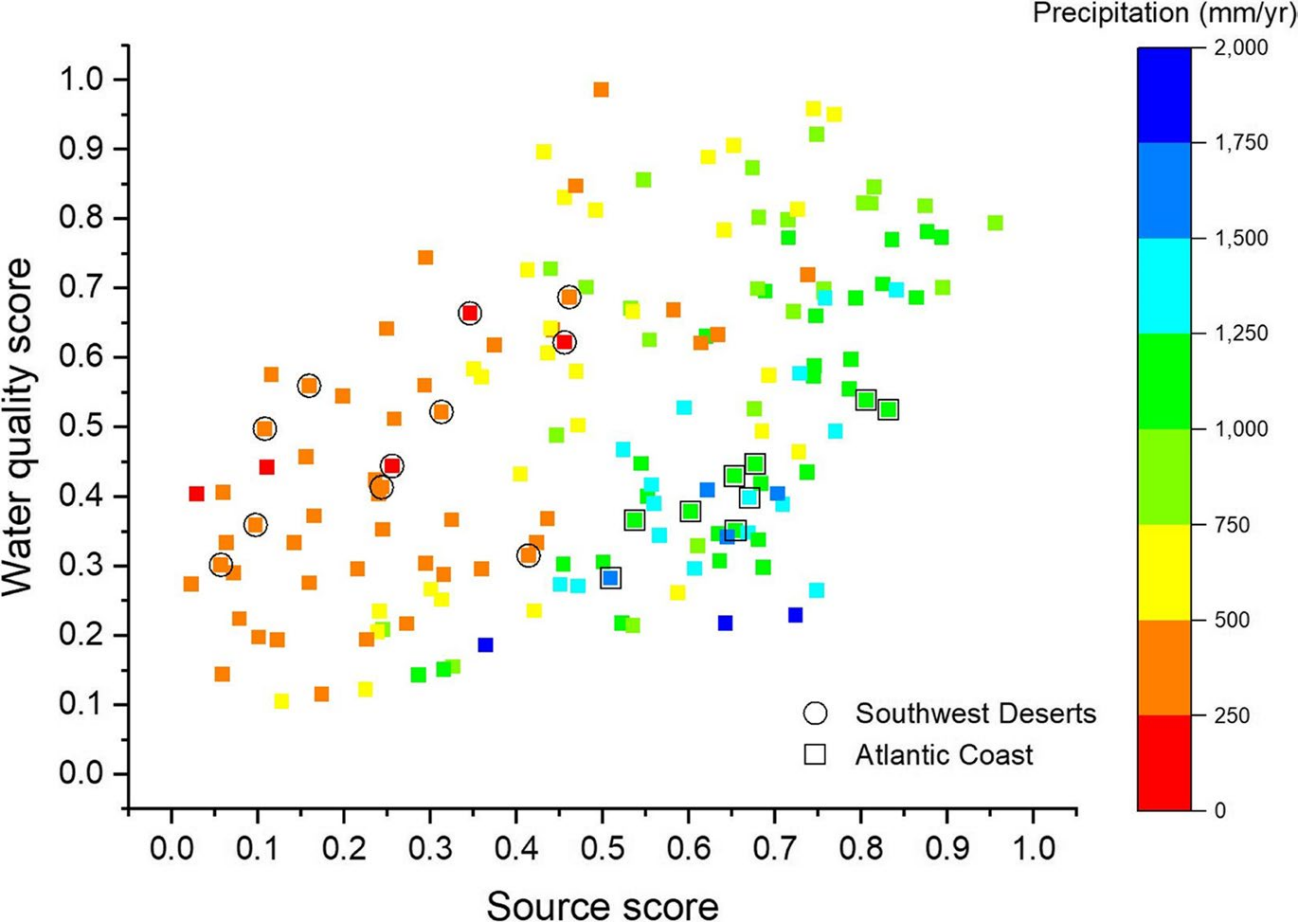
Plot of source scores versus water quality scores for the impact ranking approach. Symbol colors are based on the 30-year mean precipitation values for the date range of 1990–2020 (PRISM, 2021). Basin scores for the Southwest Deserts and Atlantic Coast regions are outlined with circles and squares, respectively, to illustrate regions with low and high precipitation rates, respectively

In the current study, a comparison of the national source scores to the water-quality scores shows that relatively low precipitation basins (PRISM Climate Group 2021), such as many in the Texas, Central Rockies, and Southwest Deserts regions, tended to have lower source scores for a given water-quality score than HRs with high productivity like  many basins the Atlantic Coast and the Pacific Northwest regions (Fig. 5). Such a pattern may reflect a combination of factors including less primary productivity (nutrient uptake) and less dilution in arid regions compared to more humid regions.

The highest receptor scores were in many of the same HRs as the highest source scores (Fig. 3). In the California-Nevada region (HR 18), the high receptor score for the San Francisco Bay basin contributed to it becoming the second most highly ranked basin in this HR. High nutrient inputs and nutrient enrichment effects in the San Francisco Bay basin are well documented, with the potential for increased enrichment effects in the coming decades (Cloern and Jassby 2012, Cloern et al. 2020).

The scores for the variability approach for the CONUS (Fig. 4) reflect the emphasis on ranges, rather than means or medians of variables within basins. The Northeast (HR 1), Great Lakes (HR 4), Columbia-Snake (HR 14), and California-Nevada (HR 18) regions were among the basins that tended to have the highest water-quality scores, and the Midwest (HR 5) and Souris-Red-Rainy (HR 9) regions were among the basins with low scores (Fig. 4, top panel). The low scores for the Midwest and Souris-Red-Rainy HRs reflect the lack of HUC08 subbasins with low nutrient concentrations in these regions. The areas on the eastern side of the U.S., the Northeast (HR 1), Atlantic Coast (HR 2), Florida (HR 3), Great Lakes (HR 4), Midwest (HR 5), and Tennessee-Missouri (HR 6) often had high scores for the factors affecting the nutrient concentrations category, while basins in the Northern High Plains (HR 10), Central Rockies (HR 15), and Southwest Desert (HR 16) regions tended to have lower scores (Fig. 4, second panel). For model accuracy, basins in HRs just east of the Rockies, such as the Northern High Plains (HR 10), Central High Plains (HR 11), and Southern High Plains (HR 12) regions, often had high scores while basins in the Atlantic Coast (HR 2) and Pacific Northwest (HR 17) often had low scores. Similar to the range of factors affecting nutrient concentrations, basins in the HRs on the eastern side of the U.S. (HRs 1–8) often had high scores in the receptor category, while basins in the Northern High Plains (HR 10), Central Rockies (HR 15), and Southwest Desert (HR 16) regions often had low scores (Fig. 4, bottom panel).

### Ranking within each hydrologic region

**Fig. 6:**
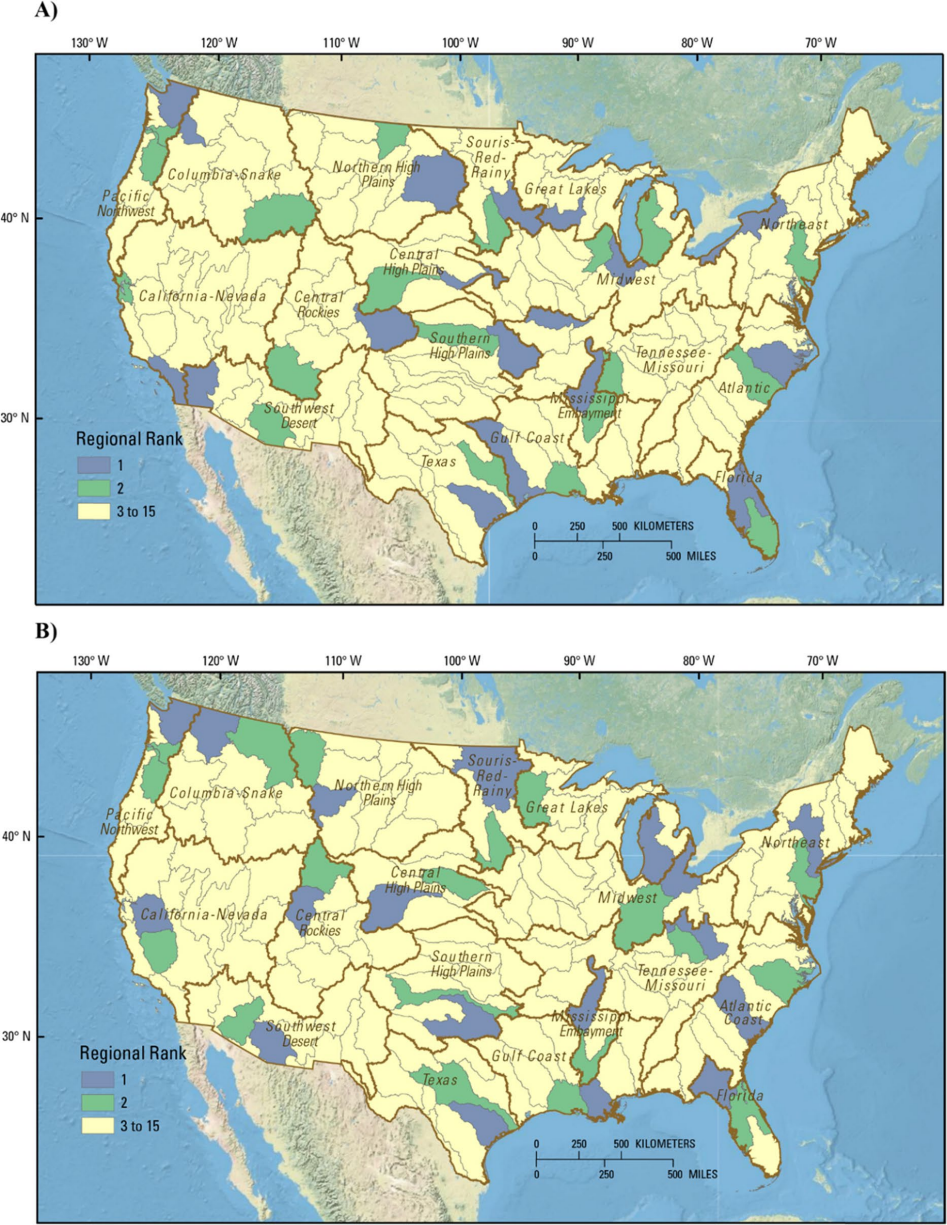
Top-ranked candidate basins for each hydrologic region throughout the contiguous United States for (A) impact ranking and (B) variability ranking. The names of the top-ranked basins are provided in Table 3. Ranking and supporting data are provided in the Supplemental material and in Qi et al. (2023)

**Fig. 7:**
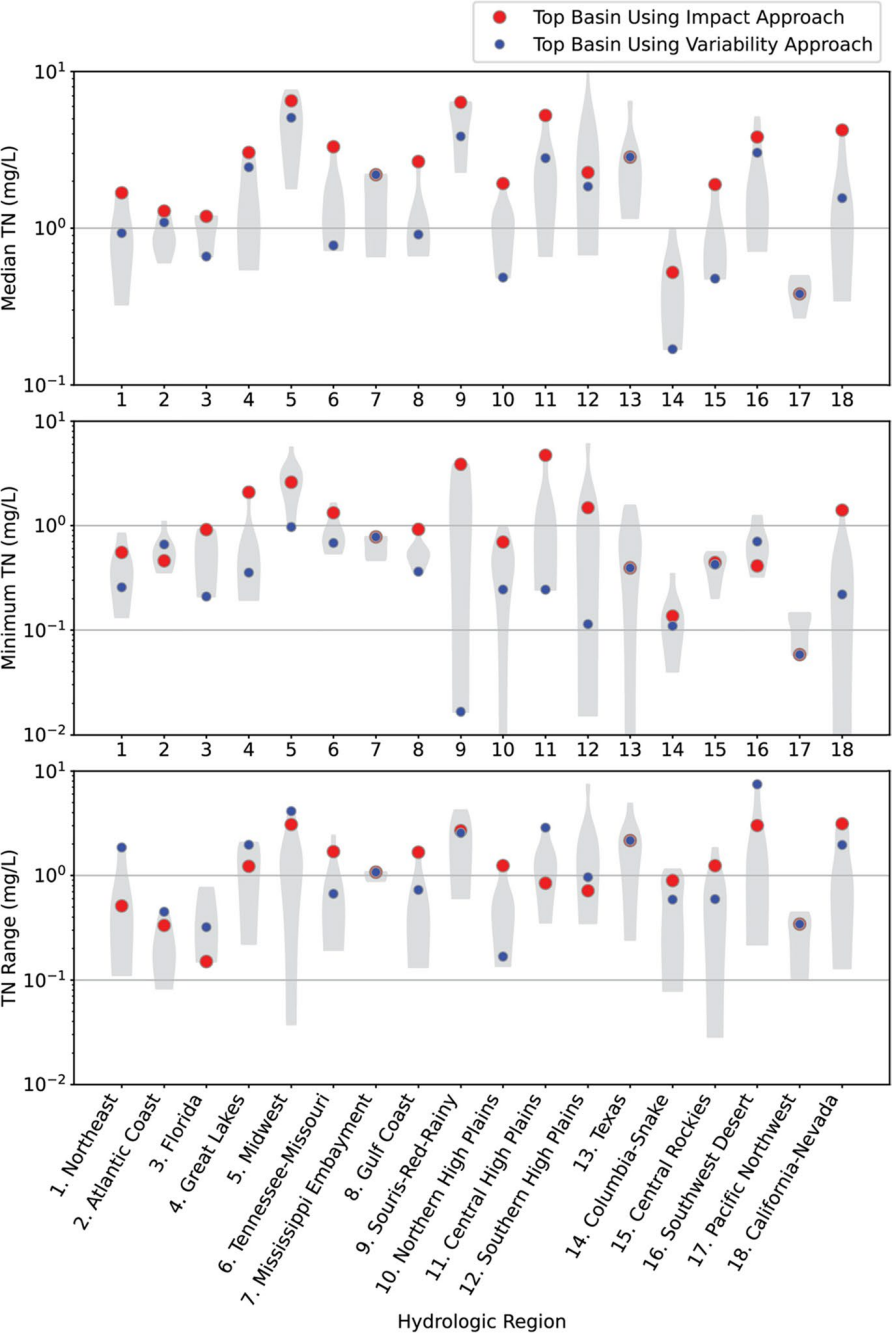
Distribution of median predicted total N concentration (top), minimum predicted total N concentration (middle), and range in predicted total N concentration for HUC08-sized basins within candidate basins in each region. Distributions are shown using violin plots. Violin plots depict data distributions using density curves; the width of a curve corresponds to the frequency of data at a point. The overall top ranked basins for the impact and variability approaches are identified with red and blue dots, respectively

The overall ranking within each hydrologic region (HR) was used to reduce the number of candidate basins for nutrient studies from 163 to 36, assigning the top 2 candidate basins in each HR as priority basins (Table 3 and Fig. 6). The selection of the top two basins within each HR was performed using both the impact and variability approaches. In 15 of the 18 HRs, a different basin was selected as the top choice by the variability approach than was chosen by the impact approach (Table 3). In the HRs where the top selections differ, the way these approaches incorporate water quality was a major driving factor. For example, note the large differences in the water-quality scores between the top-ranked basins using the impact approach and the variability approach in HRs of the Northeast (1), Florida (3), Great Lakes (4), Tennessee-Missouri (6), Gulf Coast (8), Souris-Red-Rainy (9), Central High Plains (11), Southern High Plains (12), Southwest Desert (16), and California-Nevada (18) (spread between blue and red dots in Fig. 4, top panel). Differences in the scores for the other three categories were generally much smaller (see bottom three panels in Fig. 4). Modeled stream nutrient concentrations in the selected basins provided important insight into the kinds of basins selected using the impact and variability approaches. The top-ranked basins using the impact approach tended to have higher median N and P concentrations and higher minimum N and P concentrations than the top-ranked basins with the variability approach (Fig. 7, Fig. S-1). The range in N and P concentrations also tended to be greater for the top-ranked basin from the variability approach than the top-ranked basin from the impact approach, but the differences were smaller than for the median and minimum values.

**Table 3:** Top-ranked basins in each region determined using the impact and variability approaches

Region	Top-ranked basins Impact approach	Top-ranked basins Variability approach	Region number	Region rank
Northeast	Lake Erie and Ontario	Hudson	1	1
	Delaware	Delaware	1	2
Atlantic Coast	NC eastern	Edisto-Santee	2	1
	Pee Dee	NC eastern	2	2
Florida	Florida northcentral	Suwannee	3	1
	Southern Florida	Florida northcentral	3	2
Great Lakes	Upper Mississippi-Black-Root	Eastern Lake Michigan	4	1
	Eastern Lake Michigan	Mississippi Headwaters	4	2
Midwest	Upper Illinois	Western Lake Erie	5	1
	Rock	Wabash	5	2
Tennessee-Missouri	Lower Missouri	Ohio-Big Sandy-Guyandotte	6	1
	Lower Mississippi-Hatchie	Kentucky-Licking	6	2
Mississippi Embayment	Lower Mississippi-St. Francis	Lower Mississippi-St. Francis	7	1
	Lower Mississippi-Yazoo	Boeuf-Tensas-Big Black	7	2
Gulf Coast	Trinity-San Jacinto	Lower Mississippi	8	1
	Louisiana Coastal	Louisiana Coastal	8	2
Souris-Red-Rainy	Minnesota	Red	9	1
	Missouri-Big Sioux	Missouri-Big Sioux	9	2
Northern High Plains	Missouri-Oahe	Upper Yellowstone	10	1
	Missouri-Poplar	Missouri-Marias	10	2
Central High Plains	Platte	South Platte	11	1
	South Platte	Loup	11	2
Southern High Plains	Neosho-Verdigris	Red-Washita	12	1
	Middle Arkansas	Lower Canadian	12	2
Texas	Central Texas Coastal	Central Texas Coastal	13	1
	Lower Brazos	Lower Colorado-San Bernard Coastal	13	2
Columbia-Snake	Yakima	Upper Columbia	14	1
	Upper Snake	Kootenai-Pend Oreille-Spokane	14	2
Central Rockies	Upper Arkansas	Lower Green	15	1
	Little Colorado	Great Divide-Upper Green	15	2
Southwest Desert	Southern Mojave-Salton Sea	Middle Gila	16	1
	Middle Gila	Lower Gila	16	2
Pacific Northwest	Puget Sound	Puget Sound	17	1
	Willamette	Willamette	17	2
California-Nevada	Southern California Coastal	San Joaquin	18	1
	San Francisco Bay	Tulare-Buena Vista Lakes	18	2

Ranking and supporting data are provided in the supplemental material and in Qi et al. (2023)

A comparison of the top selections in the Souris-Red-Rainy region (HR 9) provides an illustration of how the two approaches may diverge in areas with high nutrient applications. The Minnesota River basin was the top-ranked basin in this HR using the impact approach, while the Red River basin was the top-ranked basin using the variability approach. The Minnesota River basin is a major contributor of N to the Mississippi River (David, Drinkwater and McIsaac 2010) and the Gulf of Mexico (Goolsby and Battaglin 2001). While these high inputs to the Mississippi River and the Gulf of Mexico may give this basin high importance with respect to improving nutrient management, the lack of low nutrient concentrations throughout its subbasins (Minnesota River Basin Data Center 2009) may make it difficult to conduct studies that seek to understand the causes of high nutrient concentrations and the impacts of nutrients on stream ecosystems. The lack of low nutrient concentrations and lack of variability in concentrations in its subbasins contributed to the Minnesota River basin being ranked lower than the Red River basin using the variability approach. While the Red River basin has significant agricultural land use, it also has a greater percentage of forest land than the Minnesota River basin (Kelly et al. 2017). As a result of this more diverse land use, less impacted subbasins are more common in the Red River basin than in the Minnesota River basin.

### Advantages of impact and variability ranking approaches

The impact ranking approach, similar to an approach previously used to choose areas for intensive study (Van Metre et al. 2020), prioritizes the more stressed basins in terms of human development and nutrient concentrations. Therefore, the impact approach should identify locations where actions to reduce nutrient impacts on water quality may have the largest effect on ecosystems and human health. However, these highest ranked basins identified with the impact approach may have high nutrient concentrations throughout their basins and may have relatively homogeneous environmental factors forcing these high concentrations. This lack of variability can make it difficult to understand which factors resulted in elevated nutrient concentrations and how nutrient concentrations affected stream ecology. Most studies that have successfully linked the impact of nutrients on stream ecology have shown that the largest changes and effects occur at relatively low nutrient concentrations (Robertson et al., 2006; Wang et al., 2007). When nutrient concentrations in streams are uniformly high, other factors (e.g., light) may limit ecological responses, such that nutrient saturation occurs (Munn, Frey and Tesoriero 2010, Munn et al. 2018). As a result, the causes of the ecological effects may be difficult to determine if all nutrient concentrations in a study area are near or above nutrient saturation, especially in empirical or statistically based studies.

In contrast to the impact approach, the variability approach prioritizes basins that have a range in nutrient concentrations, lower nutrient concentrations, and a range in the factors affecting nutrient concentrations. Thus, the higher ranked basins identified using the variability approach, if studied in detail with surface-water and groundwater models, should provide more information to understand the factors affecting surface water quality and how water quality may affect ecosystems. This information would provide more statistical power to refine and calibrate water-quality models used at local and national scales. Basins that are highly ranked by both ranking approaches may be suitable for both identifying locations where actions to reduce nutrient impacts on water quality would have the largest effect and for conducting studies that identify the factors affecting surface water quality and ecosystem health.

## Limitations and future improvements in ranking approaches

Ranking basins using either the impact or variability approach has several limitations. When data have a limited range, small differences in a particular variable (e.g., predicted N and P concentrations in streams), especially over a relatively small area, can result in large differences in ranking. This effect can be evaluated by examining the underlying values that contribute to a particular score or ranking. Reliance on modeled concentrations also introduces some uncertainty. For example, SPARROW predictions, which were used to calculate N and P concentrations in streams and loads to receiving waters, may have relatively large errors in some areas (Robertson and Saad 2019). These mean errors for specific areas were, however, explicitly included in the variability ranking. Another limitation is that the ranking with the variability approach may be dependent on the scale used to evaluate variability, which was at the HUC08 scale in this study. The variability in water quality being evaluated in the ranking should optimally be determined by the size of sites that would potentially be examined in future studies.

The way in which reservoirs are included in the current ranking approaches is relatively simplistic. River and reservoir interactions are often integral to water quality and river health and are simply characterized in the present analysis. In particular, small water bodies, such as ponds and impoundments, may have substantial effects on nutrients that influence river health. The present analysis could be improved by directly accounting for reservoirs as receiving waters of nutrient loads, rather than using the percentage of the basin with open water.

Lastly, the true impacts of nutrients are often hard to quantify. Nutrient concentrations and loads are in some cases only proxies for the concerns of stakeholders (e.g., potential for harmful algal blooms), while in other cases high nutrient concentrations (e.g., some drinking water supplies) may be mitigated by water treatment. In addition to potential direct human health (Ward et al., 2018) and ecosystem effects (e.g., ammonia toxicity to fish), nutrient concentrations may only be indirect indicators of harm. A primary concern for ecosystems is the extent of eutrophication that describes a range of conditions encompassing multiple drivers of excessive algal and macrophyte growth. Periods of imbalance may arise where algal communities favor assemblages that produce harmful toxins or where algal and macrophyte senescence drives overconsumption of dissolved oxygen, which may result in hypoxia and the suffocation of macrofauna and fish kills (Jenny et al. 2016, Rabalais et al. 2010, Rao et al. 2014). A challenge for nutrient-based evaluations of priority basins is recognizing that ecological health may become endangered at moderate or even relatively low nutrient concentrations. In this regard, the variability approach may put more focus on basins in transition toward increasing harm of eutrophication.

## Conclusions

Two systematic, quantitative approaches were developed to prioritize river basins for nutrient studies. The impact approach relied on 14 geospatial variables representing surface-water and groundwater nutrient concentrations, sources of N and P, and potential and observed receptor impacts. The variability approach relied on 20 geospatial variables, many that were in the impact approach, but placed greater emphasis on assuring that a range of nutrient concentrations and factors affecting nutrient concentrations occurred in subbasins within highly ranked basins. The variability approach also favored basins with high surface-water quality model uncertainty. Highly ranked basins determined from the impact approach were concentrated in agricultural areas of the Midwest region, while highly ranked basins using the variability approach were more dispersed throughout the country. The final choice of basins for future study, therefore, may benefit from considering the objectives of the study in the context of information from both approaches. One strategy to prioritize study areas would be to use a combination of the impact and variability approaches. For example, the variability ranking approach could be used first to find the best locations to implement studies to better understand the factors affecting water quality and how nutrient concentrations affect ecosystems. Management-action studies could then be conducted in the highest ranked basins identified using the impact approach where actions may have the largest effect.

### Supplementary Information

Below is the link to the electronic supplementary material.

Supplementary Fig. S1 Distribution of median predicted total P (TP) concentration (top), minimum predicted total P concentration (middle), and range in predicted total P concentration for HUC08-sized basins within candidate basins in each region. Distributions are shown using violin plots. Violin plots depict data distributions using density curves; the width of a curve corresponds to the frequency of data at a point. The overall top ranked basins for impact and variability approaches are identified with red and blue dots, respectively (EPS 3.62 MB)

Supplementary file2 (XLSX 144 KB)

Supplementary file3 (XLSX 167 KB)

## Data Availability

Data used in this study are included in the Supplemental information provided with this published article and in an associated data release (Qi et al. 2023).
